# Lasso technique for bilateral pulmonary arterial banding

**DOI:** 10.1093/icvts/ivaf130

**Published:** 2025-06-04

**Authors:** Hisayuki Hongu, Koji Nomura, Izumi Hamaya, Shinya Ugaki, Toshikazu Shimizu, Masaaki Yamagishi, Eijiro Yamashita

**Affiliations:** Department of Cardiovascular Surgery, Saitama Children’s Medical Center, Saitama, Japan; Department of Cardiovascular Surgery, Saitama Children’s Medical Center, Saitama, Japan; Department of Cardiovascular Surgery, Saitama Children’s Medical Center, Saitama, Japan; Department of Cardiovascular Surgery, Saitama Children’s Medical Center, Saitama, Japan; Department of Cardiovascular Surgery, Saitama Children’s Medical Center, Saitama, Japan; Department of Pediatric Cardiovascular Surgery, Children’s Medical Center, Kyoto Prefectural University of Medicine, Kyoto, Japan; Department of Pediatric Cardiovascular Surgery, Children’s Medical Center, Kyoto Prefectural University of Medicine, Kyoto, Japan

**Keywords:** bilateral pulmonary arterial banding, lasso technique, Fontan candidate, aortic arch obstruction, persistent truncus arteriosus

## Abstract

**OBJECTIVES:**

Bilateral pulmonary arterial banding (bil.PAB) is used as the initial palliative operation for patients with univentricular and biventricular physiology, particularly in smaller patients and in those with multiple comorbidities. Our goal was to report the midterm results of the lasso technique for bil.PAB.

**METHODS:**

The bilateral pulmonary artery (PA) was encircled with a lasso created using a Gore-Tex suture CV-4. The banding diameter was adjusted via a tourniquet using transoesophageal echocardiography to achieve a luminal diameter of 1.5 mm. From 2017 onward, 55 consecutive patients underwent bil.PAB via this technique.

**RESULTS:**

Median age/body weight was 7 days/2.9 kg, and 21/34 patients exhibited biventricular physiology/univentricular physiology, respectively. The median follow-up period was 2.7 years. The median luminal diameter and flow velocity of the right/left PA at the banding site were 1.4/1.4 mm and 3.0/3.3 m/s, respectively. Readjustment was required in 7 cases, all involving further tightening. The median interval between banding and de-banding was 3.0/1.2 months (biventricular/univentricular). Upon de-banding, adequate dilation was achieved after lasso removal and bougie dilation. During follow-up, 11 patients (20%) required PA augmentation for a hypoplastic central PA. Only 2 cases required surgical augmentation at the banding site in the late or interstage phase.

**CONCLUSIONS:**

The lasso technique is technically simple and allows fine adjustments in bil.PAB. A narrower banding width reduces residual stenosis and supports PA growth.

## GRAPHICAL ABSTRACT

**Figure:**
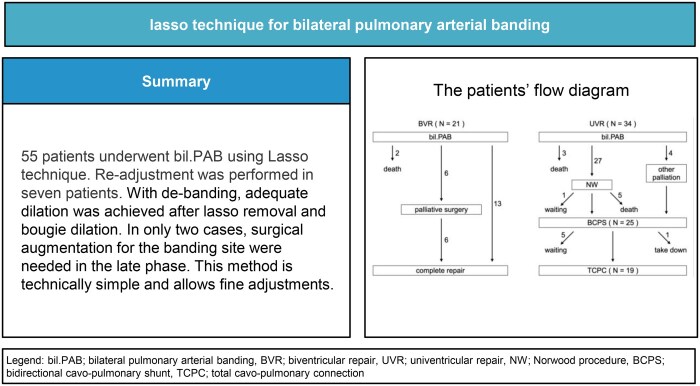

## INTRODUCTION

Bilateral pulmonary arterial banding (bil.PAB) has been established as the initial palliative operation for univentricular repair in patients with hypoplastic left heart syndrome (HLHS) and its variants [1–5]. Currently, bil.PAB has also shown benefits as a bridge to biventricular repair in patients with biventricular circulation or borderline left ventricle size, especially in small patients and those with multiple co-morbidities [6, 7]. Bil.PAB, a simple operative technique, stabilizes neonates with pulmonary overcirculation and duct-dependent blood flow in the immediate postnatal period. It avoids exposure to cardiopulmonary bypass in high-risk neonates, thereby decreasing mortality and improving overall survival [8–10]. However, bil.PAB has been reported to compromise pulmonary arterial growth potential and is considered a risk factor for residual pulmonary artery (PA) stenosis after de-banding, often necessitating additional PA interventions [11, 12]. Since 2017, we have adopted the lasso technique for bil.PAB to overcome this drawback. This technique allows fine and precise adjustment of the PA band intraoperatively, allowing for an easier, less invasive readjustment procedure. We report the mid-term outcomes of the lasso technique.

## MATERIALS AND METHODS

### Ethical statement

This study was approved by the institutional review board of the Saitama Children’s Medical Center (Approval No. 2024-04-001; 14 November 2024). It was a retrospective observational study, so it was not necessary to obtain written or verbal consent. For outpatients who wish to be informed about the research, the researcher provides an oral explanation, and the details are recorded in the patient's medical record. For other patients, a notice is posted on the hospital’s website, allowing them the opportunity to decline participation. Collection and storage of data from research participants for multiple and indefinite use strictly comply with requirements outlined in the World Medical Association Declaration of Taipei. The institutional review board approved the establishment and continuously monitors ongoing use of the database.

### Data collection and patient characteristics

Clinical data were obtained by retrospectively reviewing medical records, operative notes and echocardiographic and catheterization reports. This study focused on mortality, late death, banding readjustment, surgical reintervention for central PA and details of banding (banding circumference, lumen diameter and flow velocity at the banding site in the operating room), as well as the transition of the Nakata index [13]. Given the differing operative strategies between the univentricular physiology and the biventricular physiology groups, parts of the analyses were conducted separately. The last follow-up date was defined as the most recent outpatient visit or the date of death until April 2024. For patients in the biventricular physiology group, the Nakata index after the complete repair utilized the most recent catheterization report. In univentricular physiology, the Nakata index was measured at the pre-bidirectional cavopulmonary shunt (BCPS), the pre-total cavopulmonary connection (TCPC) and the latest examination post-TCPC.

### Statistical analyses

Statistical analyses were performed using JMP software version 13.2.0 (SAS Institute, Cary, NC, USA). Categorical variables are expressed as frequencies and percentages. Continuous data are presented as medians with interquartile ranges. The normal distribution of continuous variables was assessed via the Shapiro-Wilk test. Variables were compared using the Mann–Whitney *U*-test because the values reported in this study, including body weight, age, banding circumference, luminal diameter, flow velocity and Nakata index, did not follow a normal distribution. The Nakata index for each group was analysed using paired tests. For missing data, missingness was assumed to be completely random, and a complete case analysis method was applied. The percentage of missing data was less than 5%, making its impact on loss of precision and bias negligible. Follow-up time was estimated using the simplified person-time method [14].

### Lasso technique

**Figure 1:**
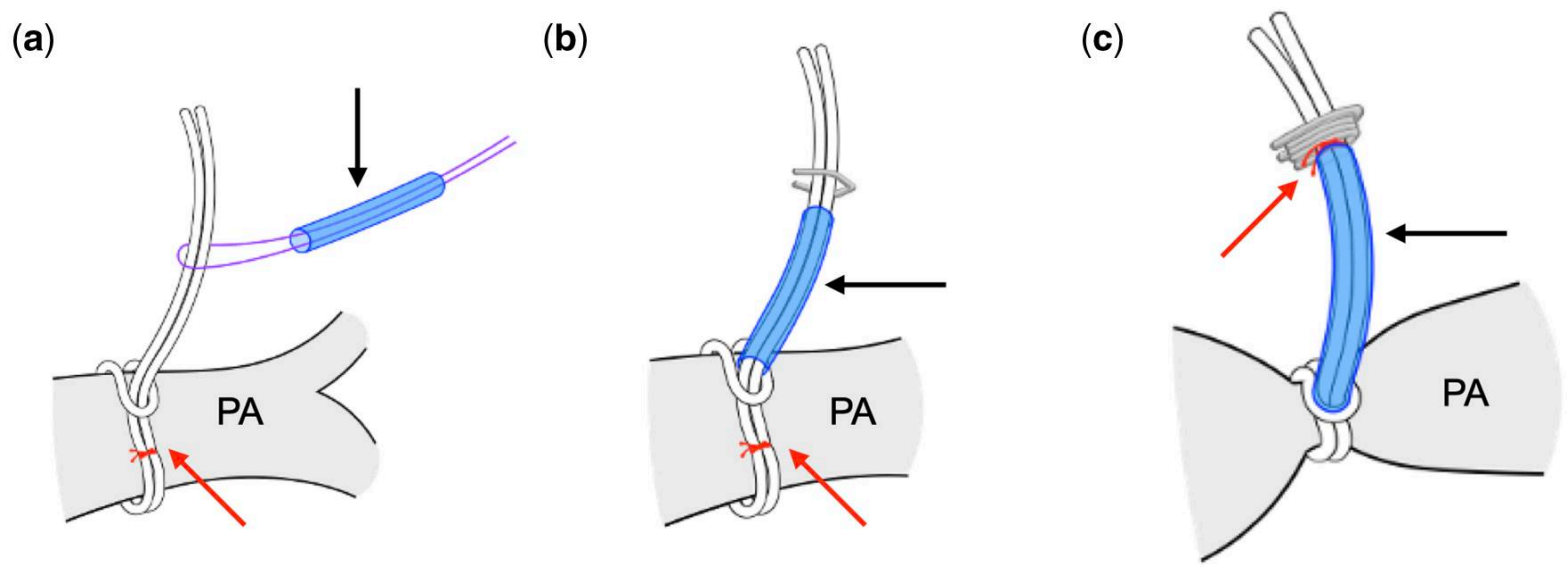
Details of the lasso technique. (**a**) The right/left pulmonary artery is encircled with a Gore-Tex CV-4 suture in a lasso fashion. The downward pointing upper arrow shows a 10-mm tourniquet made with a 4 Fr polyvinyl chloride tube. The lower arrow shows the marking suture. (**b**) After applying a tourniquet (upper arrow), vascular clips were attached or removed just above the tourniquet to adjust the banding diameter. The lower arrow points to the marking suture. (**c**) A vascular clip is attached to the marked position, resulting in a pulmonary artery circumference of 12 mm. The arrow points to the marking suture. PA: pulmonary artery

**Figure 2:**
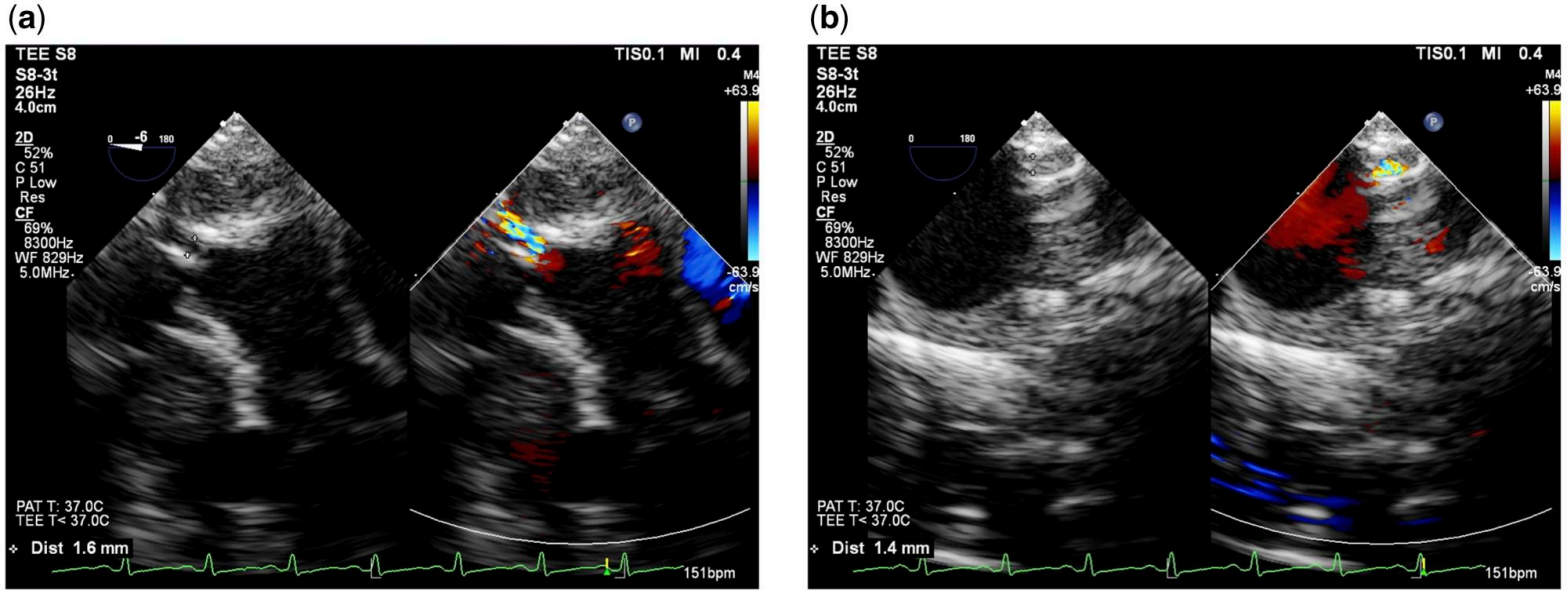
Adjustment of the bilateral pulmonary artery using transoesophageal echocardiography. The target lumen diameter was about 1.5 mm. (**a**) Right pulmonary artery after banding. (**b**) Left pulmonary artery after banding.

The lasso was created using Gore-Tex suture CV-4 (W.L. Gore & Associates, Flagstaff, AZ, USA). The CV-4 suture was folded in half preoperatively (Fig. 1), and a marking suture was placed for the CV-4 sutures 22 mm from the fold. During the operation, the bilateral PA encircled the CV-4 suture in a lasso-like fashion. A 10-mm long tourniquet made of 4 Fr polyvinyl chloride tubing was applied to each lasso. Vascular clips were attached to or removed immediately above the tourniquet to adjust the banding diameter. Initially, a vascular clip was attached to the marked position, and the PA circumference was calculated as 12 mm. If the banding required strengthening, an additional clip was attached between the currently attached clip and the tourniquet. If loosening was necessary, the clips were removed individually. This configuration was adjusted using transoesophageal echocardiography to achieve a lumen diameter of 1.5 mm at the banding site (Fig. 2). Finally, the banding diameter was determined based on the patient’s oxygen saturation, haemodynamic and arterial blood gas analysis results. If necessary, epicardial echocardiography was performed. In typical cases, when the circumference is adjusted to around 11 mm (diameter 3.5 mm), the lumen diameter is approximately 1.5 mm, and fine adjustment is rarely required. Although, in unusual cases with special haemodynamics, such as patients with low body weight and severe atrioventricular valve regurgitation that is difficult to manage, and patients with HLHS with an intact interatrial septum, fine adjustments may be needed. Thus, all measurements, including banding circumference, lumen diameter and flow velocity at the banding site, were recorded during the procedure. Because the ductus arteriosus must remain unaffected by the banding string, evaluating its morphology before the procedure was essential. Furthermore, this evaluation was consistently performed during the operation using transoesophageal echocardiography. The efficacy of bil.PAB was notably influenced by haemodynamic parameters, including intravascular volume, blood pressure and heart rate. Given that band tightness can change during the operation and the perioperative period, real-time haemodynamic observations were essential to determine whether further tightening or loosening was required.

## RESULTS

### Patient characteristics

From January 2017 to April 2024, a total of 55 consecutive patients underwent bil.PAB using the lasso technique. Of these, 21 had biventricular physiology and 34 had univentricular physiology. In the biventricular physiology group, 17 patients were diagnosed with aortic arch obstruction complex (coarctation of the aorta or interrupted aortic arch), 3 patients were diagnosed with patent truncus arteriosus and 1 patient was diagnosed with a large patent ductus arteriosus, double-outlet right ventricle, pulmonary atresia, complete atrioventricular septal defect and severe atrioventricular valve regurgitation. In the univentricular physiology group, 12 patients were diagnosed with HLHS; 10, with the HLHS variant; and 12, with other single-ventricle diseases. The median age and body weight at the time of the operation were 7 days (IQR 3.5–11.5) and 2.9 kg (IQR 2.6–3.2), respectively (Table 1).

**Table 1:** Patient characteristics

	Diagnosis		Age	Body weight
All	55		7 days (IQR 3.5–11.5 days)	2.9 kg (IQR 2.6–3.2 kg)
Biventricular repair (*N* = 21)	AAO complex PTA Other	17 3 1		
Univentricular repair (*N* = 34)	HLHS HLHS variant Others	12 10 12		

AAO: aortic arch obstruction; HLHS: hypoplastic left heart syndrome; IQR: interquartile range; PTA: patent truncus arteriosus.

### Outcome

The median follow-up period was 2.7 years (IQR 1.5–4.9). Early death and late death were observed in 2 and 8 patients, respectively. Early death was attributed to low output syndrome. Among the 8 patients, the causes of late death were identified as sepsis (*n* = 2), arrhythmia (*n* = 3), chronic heart failure (*n* = 1), low output syndrome (*n* = 1) and unknown reasons (*n* = 1).

### Banding details in the operating room

The median banding circumference was 11.0 mm (IQR 10.0–11.0) in the right PA and 11.0 mm (IQR 11.0–11.0) in the left PA. The median lumen diameter at the banding site, measured by transoesophageal echocardiography, was 1.4 mm (IQR 1.3–1.7) in both the right and left PA. In addition, the median flow velocity at the right and left banding sites was 3.0 m/s (IQR 2.8–3.4) and 3.3 m/s (IQR 3.0–3.6), respectively (Table 2).

**Table 2:** Details of results of bilateral pulmonary arterial banding

	rt.PA	lt.PA
Median banding circumference (mm)	11.0 (IQR 10.0–11.0)	11.0 (IQR 10.0–11.0)
Median lumen diameter (mm)	1.4 (IQR 1.3–1.7)	1.4 (IQR 1.3–1.7)
Median flow velocity (m/s)	3.0 (IQR 2.8–3.4)	3.3 (IQR 3.0–3.6)
Readjustment case	7 (tighten: 7)	

IQR: interquartile range; lt: left; PA: pulmonary artery; rt: right.

### Readjustment

Seven cases required banding readjustment, with all cases needing tightening. In all 7 cases, the luminal diameter at the banding site was not loose. The procedures for loosening the banding, including percutaneous transluminal angioplasty, were not identified. The median interval between bil.PAB and the readjustment procedure was 3 days (IQR 2.5–7.0). In all readjustment cases, flow regulation was achieved by simply adding a vascular clip between the currently attached clip and the tourniquet.

### Patient developments

**Figure 3:**
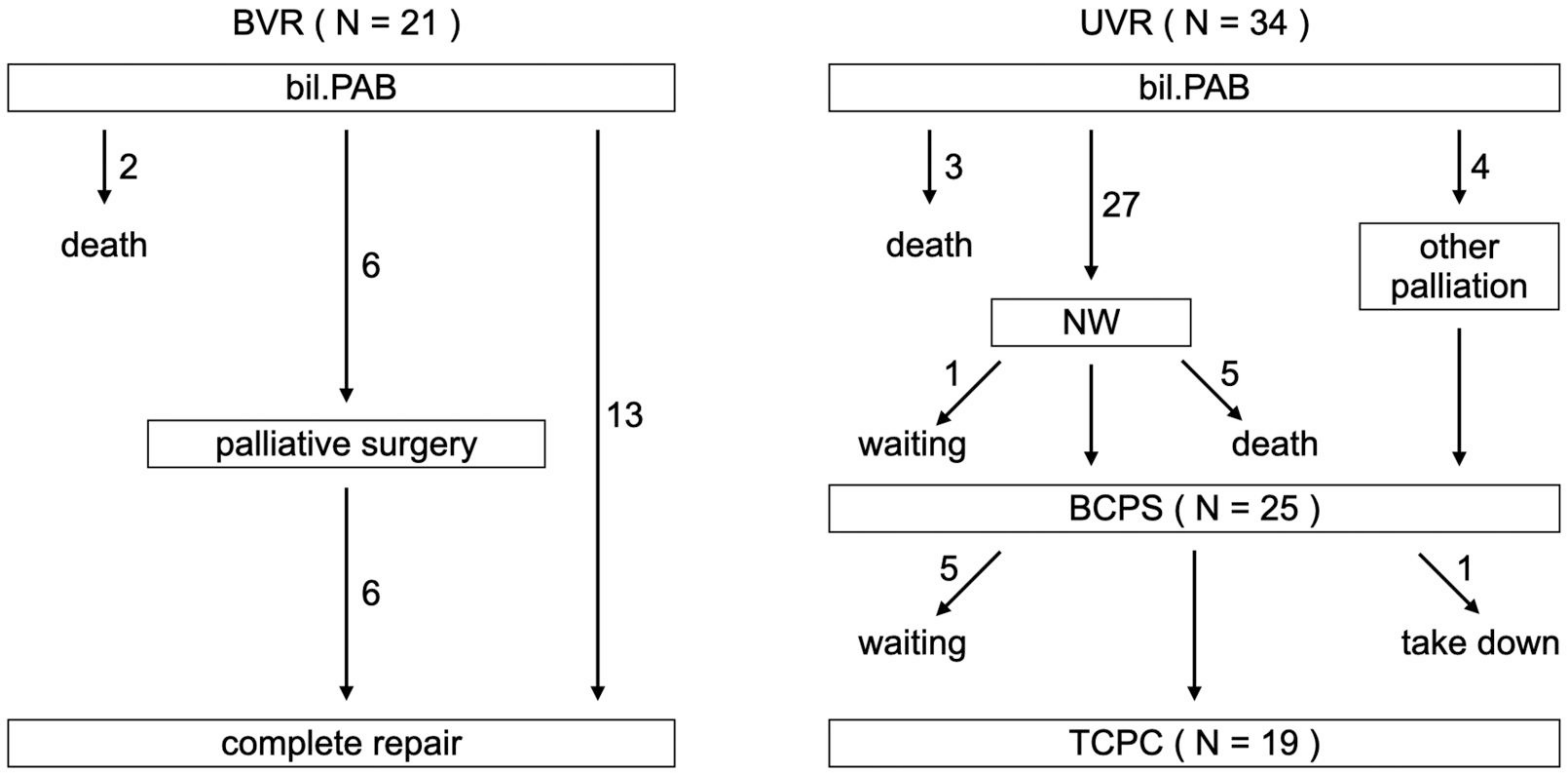
The patients’ flow diagram. BCPS: bidirectional cavopulmonary shunt; bil.PAB: bilateral pulmonary arterial banding; BVR: biventricular repair; NW: Norwood procedure; TCPC: total cavopulmonary connection; UVR: univentricular repair

A patient flow diagram is shown in Fig. 3. In the biventricular physiology group, the median interval between bil.PAB and the de-banding procedure was 3.0 months (IQR 2.1–3.9). When the de-banding procedure was performed, adequate dilation was achieved in all cases with the removal of the banding lasso and bougie dilation alone. None of the patients required surgical PA augmentation with supplementary material. In the late phase, only 1 patient required surgical augmentation of the banding region. The most recent cardiac catheterization procedure demonstrated that the Nakata index was 198.3 mm^2^/m^2^ (IQR 159.3–276.8).

In the univentricular physiology group, the median interval between bil.PAB and the de-banding procedure was 1.2 months (IQR 1.0–1.7) (biventricular physiology vs univentricular physiology; *P* = 0.0001). In 1 case, the right PA banding migrated to the peripheral PA branches. In this case, catheter intervention was effective; therefore, surgical augmentation of the PA branches was not required. With the de-banding procedure, adequate dilation was achieved in all cases, with only the removal of the banding strip and bougie dilation. None of the patients required augmentation with supplementary material. Only 1 patient required surgical augmentation for the PA interstage between the Norwood procedure and the BCPS. At the BCPS, 10 patients required PA augmentation for hypoplastic central PA. In all 10 cases, 3-dimensional cardiac computed tomography was performed before BCPS, and it was confirmed that the stenotic region and the banding site were different. The Nakata index pre-BCPS, pre-TCPC and post-TCPC (the latest examination) was 127.3 mm^2^/m^2^ (IQR 107.5–164.3), 157.3 mm^2^/m^2^ (IQR 139.5–192.9) and 168.1 mm^2^/m^2^ (IQR 147.3–218.4), respectively [(pre-BCPS vs pre-TCPC, *P* = 0.008), (pre-TCPC vs post-TCPC, *P* = 0.27) and pre-BCPS vs post-TCPC, *P* = 0.001)].

## DISCUSSION

Bil.PAB was first described by McFaul *et al.* as a palliative surgical technique for patients with truncus arteriosus [15]. For univentricular candidates, Gibbs *et al.* used this procedure as part of a hybrid approach for HLHS, consisting of ductus arteriosus stenting, atrial septectomy and bil.PAB [16]. In many institutions, bil.PAB is currently used as the initial palliation of the rapid two-stage Norwood procedure for HLHS, HLHS variant and other univentricular candidates with systemic ventricle outflow tract and/or aortic arch obstruction. In addition, to delay the timing of complete intracardiac repair and aortic arch repair, bil.PAB is also utilized in patients who are biventricular candidates or whose left ventricle size is borderline, especially small patients and those with multiple co-morbidities.

In conventional procedures, a Gore-Tex patch or graft trimmed to approximately 2.0–3.5 mm wide is used as a banding tape. Simulations of fluid dynamics have demonstrated that the degree of banding on the PA diameter regulates the pressure gradient rather than the banding length [17]. Therefore, if the same banding effect is achieved, a narrower banding length is less likely to result in residual PA stenosis.

Furthermore, the lasso technique may be superior to conventional techniques in terms of the adjustability of PA banding. In conventional procedures, to tighten or loosen the banding, re-suturing of the banding tape is required on the proximal and distal sides. This procedure is often too delicate and challenging to perform in the small-sized PA of a neonate, especially in emergency cases. In contrast, the lasso technique is very useful because the banding can be adjusted simply by attaching and detaching a vascular clip. Particularly in conditions where adhesions begin to form several weeks after the operation, the superiority of the lasso technique can be realized because it does not require dissection of the PA, which is necessary with the conventional technique. In the 7 cases of readjustment we experienced, dissection of the PA was unnecessary, and adjustment was possible without worsening the haemodynamics. Conventional techniques are likely to require surgical augmentation after de-banding and may cause vascular injury during de-banding. The lasso technique does not have this concern.

With this technique, an anchoring suture to secure the banding lasso to the native PA was not required. Anchoring sutures to a small PA carries the risk of unplanned PA stenosis. In our series, there was only 1 case of migration, because banding was not performed near the PA hilum bifurcation. Right PA banding should be performed on the proximal side between the ascending aorta and superior vena cava and not on the origin of the right PA to minimize dissection of the great arteries for the next operation. For left PA banding, the first branch of the left PA is often earlier than that of the right PA; therefore, we are careful to perform banding as proximally as possible. In cases where it is technically challenging to encircle the left PA that originates vertically and dorsal to the posterior wall of the main PA trunk, such as in HLHS, a traction suture to the main PA trunk on the right side can be performed. However, care must be taken not to interfere with the ductus arteriosus.

PA growth after bil.PAB has been reported in several studies. Davies *et al.* reported an increased risk of PA intervention in patients who underwent band placement exceeding 90 days [11]. Another report showed that limited growth of the PA after bil.PAB is related to the prolonged banding period [18]. This concern is more problematic in the univentricular physiology group, where the development of the PA is critical. In our study, the interval between bil.PAB and de-banding procedures was significantly shorter in the univentricular physiology group because we generally apply rapid two-stage repair for patients with a univentricular physiology. Although few reports have evaluated PA growth after bil.PAB, satisfactory PA growth was obtained compared with reports using conventional techniques, which may indicate the usefulness of the lasso technique in terms of PA growth.

The limitations of this study include its retrospective, noncomparative design at a single institution with a small sample size. Owing to this limited sample size, there may be some minor deviations in the results of the statistical comparison test between the 2 groups. Moreover, institutional bias concerning critical care, interventional management and surgical approaches should be acknowledged.

## CONCLUSION

The lasso technique is simple and easy to perform. It allows for fine adjustment of the banding diameter without worsening the haemodynamics when readjustment is required. In addition, compared with conventional procedures, the narrower banding width contributes to a lower incidence of residual stenosis in the banding region, thereby supporting favourable PA growth.

## Data Availability

The data underlying this article cannot be shared publicly to protect the privacy of individuals who participated in the study. The data will be shared upon reasonable request to the corresponding author.

## ABBREVIATIONS

BCPS: Bidirectional cavopulmonary shunt
bil.PAB: Bilateral pulmonary arterial banding
HLHS: Hypoplastic left heart syndrome
PA: Pulmonary artery
TCPC: Total cavopulmonary connection
